# The Sensitive Element of Acoustic Sensor on Circular Polarized Waves: From Theoretical Considerations towards Perspective Rotation Rate Sensors Design

**DOI:** 10.3390/s21010032

**Published:** 2020-12-23

**Authors:** Michail Shevelko, Andrey Lutovinov, Aleksandr Peregudov, Ekaterina Popkova, Yasemin Durukan, Sergey Shevchenko

**Affiliations:** Department of Electroacoustics and Ultrasonic Technology, Saint Petersburg Electrotechnical University, 197376 Saint Petersburg, Russia; mmshevelko@etu.ru (M.S.); loutovinov.ai@ultrakraft.ru (A.L.); anperegudov@etu.ru (A.P.); espopkova@etu.ru (E.P.); yadurukan@etu.ru (Y.D.)

**Keywords:** rotation rate sensor (RRS), acoustic sensor, bulk acoustic waves (BAW), circular polarized acoustic wave (CPAW), solid-state sensitive element (SE)

## Abstract

In this paper, the perspectives of using the features of acoustic wave propagation to design rotation rate sensors (RRS) are discussed. The possibility of developing the solid-state sensitive elements (SE) of RRS on acoustic waves of circular polarization is shown. The theoretical basis of bulk acoustic wave propagation under rotation is given. The direct excitation of circularly polarized acoustic wave (CPAW) is considered, the design of the CPAW emitting transducer is offered. The results of experimental studies that indicated the circular nature of the particle motions in the radiated wave are discussed. The principally new concept of the RRS SE design on CPAW, being able to operate under high vibration and acceleration, is proposed. The experimental results revealed a high correlation with theoretical and numerical predictions and confirmed RRS on CPAW operability.

## 1. Introduction

In the modern world, the RRS represent a wide class of sensors, implemented on various physical principles, used in many practical applications—from mobile phones to complex orientation and navigation systems, including ones operating in harsh vibration conditions. Also, there is a class of maneuverable objects, where the motion is characterized by ultra-large accelerations, significant roll and pitch angles. In such conditions, modern MEMS do not maintain their accuracy characteristics or even turn out to be inoperative. At the same time, the use of several types of gyroscopes built on other physical principles can be limited due to their weight and size indicators. Moreover, the disadvantage of all the mentioned types of RRS is a rather narrow dynamic range of measured angular velocities [1].

For the reasons mentioned above, an urgent scientific and industrial task is to create a fundamentally new generation of solid-state shock and vibration-resistant SE of RRS without moving inertial masses and suspension elements in its construction. The angular velocity sensors, based on acoustic waves, can be an appropriate solution.

That is the reason why interest in the research of the features of acoustic waves propagation under rotation and its use for motion parameter sensors development has increased in recent decades. Such studies have been carried out both for surface (SAW) and bulk (BAW) acoustic waves [2,3,4,5,6,7,8,9,10].

The first attempts of studying the physics of BAW propagation under rotation dates back to the 1960s. In the first work devoted to this problem [11] the propagation of BAW excited by a point source in isotropic elastic medium rotating with a constant angular velocity were considered and the motion equation were provided in terms of the vector and scalar potentials. However, as a result no specific conclusions regarding the types and velocities of propagating waves were formulated.

In [12], the BAW propagation features with a flat front under rotation were analyzed. Authors note that the presence of centripetal and Coriolis accelerations in the motion equation leads to anisotropic and dispersive medium character and hypothesized that the phase velocity of BAW depends on the ratio of the angular velocity of medium rotation and the wave radial frequency. The elliptical polarization of the waves, due to the presence of an imaginary unit in the polarization vectors ratio, is also noted. In the paper of the same group [13], a special task is considered when the direction of BAW propagation is orthogonal to the rotation axis of the medium. In this case, the types of propagating waves were found to be pure transverse, quasi-longitudinal and quasi-transverse waves, correspondingly. However, despite the general validity of the theoretical prediction of particle oscillations nature in propagating waves, the authors of [12,13] also did not provide any specific expressions for the velocities and polarization of the waves.

A similar case of BAW propagation along the direction orthogonal to the rotation axis was considered in [14]. The author made a mistaken suggestion that the polarization of the radiated wave changes, under rotation, and associated it with a change in the wave-front propagation direction, and thus, also the wave vector.

In [15] an analysis was performed of both BAW and SAW propagating in an elastic medium rotating at a constant angular velocity. The authors concluded that, under such conditions, BAW of pure linear polarization cannot propagate, and the phase velocity of SAW depends on the angular velocity of medium rotation.

Therefore, the results of theoretical studies, carried out by the mentioned authors, did not end with any practically significant conclusions and it did not lead to the development of certain principles and designs of SE on acoustic waves.

In recent years, the research group of the Department of Electroacoustics and Ultrasonic Technology of Saint Petersburg Electrotechnical University has been searching for the latest solutions in this scientific field, conducting complex theoretical, computational, and experimental studies. The characteristics of acoustic wave dependent on rotation rate were first discovered, thereby, revealing the possibility of using various types of acoustic waves in the presence of the rotation of the propagation medium for RRS SE development [16,17]. The authors proposed a number of fundamentally new physical principles and constructive solutions for RRS SE design, based on acoustic waves, where the informative parameter is the change in the characteristics of propagating acoustic waves from the rotation rate [18]. In contrast to MEMS, this type of solid-state RRS SE has no inertial masses on torsion suspensions in its design, which allows it to operate under conditions of high vibration and acceleration load. According to the authors’ estimation, the RRS SE on acoustic waves has a wider dynamic range, limited from below by inherent noise, and from above by the structural strength of SE materials.

In this way the new class of solid-state RRS SE on acoustic waves, developed by the authors, can be used in navigation and orientation systems of highly dynamic special-purpose objects, such as rocketry, aircraft, and space engineering, which has extended requirements for dynamic range and resistance to vibration and acceleration loads.

This paper represents a new approach for RRS SE design, based on circular polarized acoustic waves (CPAW).

## 2. Theoretical Analysis

### 2.1. Theoretical Basis

The theory of RRS SE is based on motion Equation (1):

(1) $$\left(\frac{\partial^{2}\xi_{i}}{\partial t^{2}}+2\left(\in_{ink}\Omega_{n}\right)\frac{\partial \xi_{k}}{\partial t}+\left(\Omega_{i}\Omega_{k}\xi_{k}-\Omega_{k}\Omega_{k}\xi_{i}\right)\right)=\frac{\partial \sigma_{ik}}{\partial x_{k}}$$

where Ω*_n_* is an angular velocity component; ρ is the medium density; ξ*_k_* is the displacement components; ∈*_ink_* is the Levi-Civita symbol; σ*_ik_* = C*_iklm_*, u*_lm_* is the mechanical stress, elastic moduli, deformation tensors, respectively; x*_k_*, *t* is the spacial and time coordinates. Equation (1) is given in tensor form.

The solution of motion Equation (1) can be found for various types of waves and its propagation medium. In the present paper Equation (1) is solved for plane harmonic waves,

$$\xi_{i}=\xi_{0i}\cdot \exp\left[j\left(\omega t-k_{m}x_{m}\right)\right],$$

where ξ_0*i*_ is an amplitude of particle displacement; *j* is the imaginary unit; ω is the radial frequency; *km* is the wave vector component.

In the present paper the material class is limited by isotropic medium. But Equation (1) solution is also valid for the BAW propagation along the crystallographic axes.

In this case the Equation (1) transforms into the Equation (2):

(2) $$\{\begin{array}{l} \left[C_{11}-\rho V^{2}\left(1+W_{2}^{2}+W_{3}^{2}\right)\right]\xi_{01}-\left(2jW_{3}-W_{1}W_{2}\right)\rho V^{2}\xi_{02}+\left(2jW_{2}+W_{1}W_{3}\right)\rho V^{2}\xi_{03}=0\\ \left[C_{44}-\rho V^{2}\left(1+W_{1}^{2}+W_{3}^{2}\right)\right]\xi_{02}+\left(2jW_{3}-W_{1}W_{2}\right)\rho V^{2}\xi_{01}-\left(2jW_{1}-W_{2}W_{3}\right)\rho V^{2}\xi_{03}=0\\ \left[C_{44}-\rho V^{2}\left(1+W_{1}^{2}+W_{2}^{2}\right)\right]\xi_{03}-\left(2jW_{2}-W_{1}W_{3}\right)\rho V^{2}\xi_{01}+\left(2jW_{1}+W_{2}W_{3}\right)\rho V^{2}\xi_{02}=0 \end{array}$$

where $W_{i}=\Omega_{i}/\omega$ is relative angular velocity; elastic moduli tensor components are given in matrix form.

Equation (2) allows determining BAW characteristics (propagation velocity and polarization type) under rotation for wave propagation along the crystallographic axes of cubic crystals and isotropic medium (C_11_ = λ + 2μ; C_44_ = μ, where λ and μ are the Lame coefficients).

The special case, when BAW is propagating along *X*_1_ axis, which is the rotational axis too, was considered by authors earlier in [16] and BAW propagation velocities were found,

(3) $$V_{1,\;2}=\sqrt{\frac{C_{44}}{\rho \left(1-W^{2}\mp 2W\right)}}$$

where *V*_1_, *V*_2_ are the propagation velocities of two waves satisfying (2).

The ratio between polarization vector components $p_{2},\;p_{3}$ can be determined by substituting *V*_1_, *V*_2_ into the Equation (2):

(4) $${\left(\frac{p_{2}}{p_{3}}\right)}_{1,2}=\pm j$$

As can be seen from the Equation (4), the oscillations along the two axes are phase-shifted by 90°, which indicates the circular polarization of the waves. Therefore, in this case, the basic waves will be CPAW with the mutually opposite direction of particle rotation.

Moreover, as seen from Equations (3) and (4), the propagation velocities and the nature of the motion of particles in the wave depend on the relative angular velocity of the acoustic duct rotation *W*.

It is known that any oscillation that is a linear displacement relative to the equilibrium can be represented as a combination of two oscillatory processes with displacements along circular paths in opposite directions around the equilibrium.

**Figure 1:**
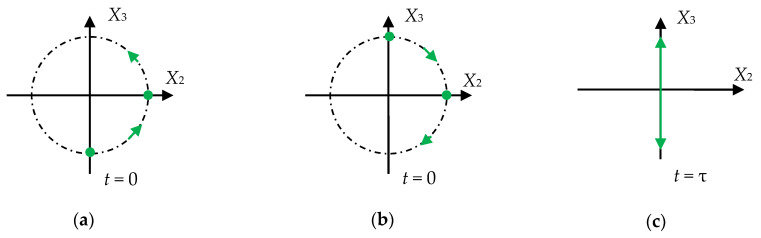
Particle movement pattern: (**a**,**b**) Basic circular polarized waves with counterclockwise, and clockwise directions of particle displacement, respectively; (**c**) total linearly polarized wave.

In this case, the nature of the particle motion in the total wave is determined by the result of adding the particle motions in two basic CPAW $\xi_{1},\;\xi_{2}$

(5) $$\xi_{1}=\xi_{y_{1}}+\xi_{z_{1}}=\xi_{0}\sin(\omega t-k_{1}x)+\xi_{0}\cos(\omega t-k_{1}x)\;\xi_{2}=\xi_{y_{2}}+\xi_{z_{2}}=\xi_{0}\sin(\omega t-k_{2}x)-\xi_{0}\cos(\omega t-k_{2}x),$$

each of which is a superposition of displacements along the axes $X_{2}$ and $X_{3}$ as shown in Figure 1.

The change (the rotation) of the particle displacement vector in the total linearly polarized wave propagating under rotation is equivalent to the excitation process of oscillations, orthogonal to the initial ones, which amplitude is proportional to the rotation rate [16].

A scientific group of authors previously suggested using this effect to create a new class of solid-state RRS. The principles of its SE construction are based on the amplitude method of revealing the informative signal and imply detecting the polarization vector rotation of the emitted transverse BAW [15,16].

### 2.2. Circularly Polarized Acoustic Waves (CPAW)

According to the expressions obtained by the authors earlier [16,17], in the presence of rotation, when the acoustic duct is exposed to centripetal and Coriolis accelerations, the phase velocity of CPAW is proportional to the rotation rate of the propagation medium, and depending on the direction of particles oscillation in waves (clockwise or counterclockwise), it either increases or decreases. As a result, when the set of CPAW propagates in a rotating medium, the displacement vector of the total linearly polarized wave rotates through an angle α proportional to the angular velocity and the wave propagation time,

$$\alpha =\frac{x}{V_{0}}\Omega ,$$

where *V*_0_ is a total wave phase velocity without rotation.

It is important to note that, despite the change in the phase velocities of the basic CPAW, the propagation velocity of the resulting wave front remains invariably equal to the phase velocity of the transverse wave in the absence of rotation. In a case where the axis of rotation of the acoustic duct is orthogonal to the direction of wave propagation, the emitted pure modes will transform into elliptically polarized ones with the compressing coefficient of the ellipse proportional to Poisson’s ratio [19].

Let us consider a plane harmonic transversal acoustic wave propagating into an isotropic half-space. The amplitude of the displacement in the wave changes according to,

ξ = ξ_0_ sin (ω*t* − k*r* + δ), (6)

where δ is the initial phase, ξ_0_ is the amplitude, ω is the angular frequency, k is the wave vector, *t* and *r* are the time and space coordinate, correspondingly.

Since the wave is transverse, and the oscillatory process takes place in an isotropic medium, the displacement vector **ξ** is perpendicular to the wave vector k.

**Figure 2:**
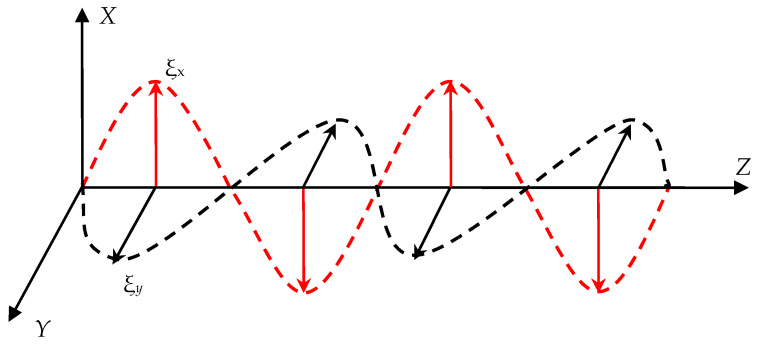
The superposition of two mutually perpendicular projections of the displacement vector ξ.

Let us consider the superposition of two mutually perpendicular projections of the vector ξ with a constant phase difference. For some certainty, assume that the wave propagates in the *Z* direction, and the projections of the vector ξ lie in the *XY* plane as shown in Figure 2.

Then:

(7) $$\xi_{x}\left(z,t\right)=\xi_{10}\;\sin(\omega t-kr)\;\xi_{y}\left(z,t\right)=\xi_{20}\sin(\omega t-kr+\delta ).$$

Equation (7) can be rewritten as follows,

$$\xi_{y}\left(z,t\right)=\xi_{20}\sin(\omega t-kr)\cos\delta +\xi_{20}\cos(\omega t-kr)\sin\delta$$

so that sin(ωt−kr) and cos(ωt−kr) can be removed and Equation (7) takes the form:

(8) $$\xi_{y}\left(z,t\right)=\xi_{20}\left(\frac{\xi_{x}}{\xi_{10}}\right)\cos\delta +\sin\delta \sqrt{1-\xi_{x}^{2}/\xi_{10}^{2}}.$$

The amplitudes $\xi_{10}$ and $\xi_{20}$ are assumed to be positive. After some mathematical manipulations Equation (8) transform into:

(9) $$\frac{\xi_{x}^{2}}{\xi_{10}^{2}}+\frac{\xi_{y}^{2}}{\xi_{20}^{2}}-2\frac{\xi_{x}}{\xi_{10}}\frac{\xi_{y}}{\xi_{20}}\cos\delta =\sin^{2}\delta .$$

**Figure 3:**
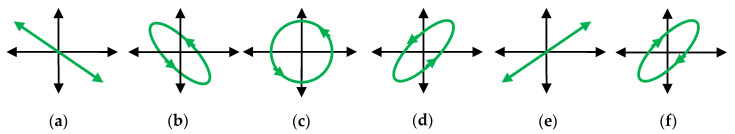
The projections of total wave on the surface, orthogonal to the wave propagation direction, for various phase shift: (**a**) phase shift δ=0, path difference $\Delta =\lambda \cdot \frac{\delta }{2\pi }$; (**b**) $\delta =\frac{\pi }{6}$ $\Delta =\frac{\lambda }{12}$; (**c**) $\delta =\frac{\pi }{2}$, $\Delta =\frac{\lambda }{4}$; (**d**) $\delta =\frac{5\pi }{6}$, $\Delta =\frac{5\lambda }{12}$; (**e**) δ=π, $\Delta =\frac{\lambda }{2}$; (**f**) $\delta =\frac{7\pi }{6}$, $\Delta =\frac{7\lambda }{12}$.

Various shapes of the projections of total wave on the surface, orthogonal to the wave propagation direction, described by Equation (9) depending on phase shift values, are shown in Figure 3.

The opposite is also true. The combination of two linearly polarized waves with a fixed phase shift gives an elliptically polarized wave. In a particular case, the superposition of two orthogonal linearly polarized waves with a phase shift of π/2 makes it possible to obtain a circularly polarized wave.

In contrast to the previous works of the authors, in this paper, it is offered to use the change in the CPAW phase velocity, while propagating in a solid-state acoustic duct along the axis of rotation, as an RRS SE informative parameter to determine the rotation rate. As a way to reveal an informative signal, phase methods are suggested, which are potentially more noiseless acoustic measurement methods. However, to implement a concept of constructing the RRS SE, it is necessary to provide the direct excitation and propagation of CPAW in a solid-state acoustic duct.

## 3. CPAW Direct Excitation

### 3.1. Theoretical Analysis

The review of literature sources [20] has shown that CPAW can be obtained, due to the interference of two purely shear waves of orthogonal polarization, with a phase difference equal to a quarter of the oscillation period. This process takes place for certain axes of anisotropic media, in which shear waves with orthogonal polarization propagate at different speeds. In this case, the emitted plane-polarized shear wave, while propagating in the medium, is transformed into an elliptically polarized one, then into a circularly polarized wave, and so on in the reverse sequence. Based on this effect, it is possible to design a transducer for CPAW direct radiation, using a buffer rod. At its one end, the transverse oscillations of linear polarization are excited with a piezo plate oriented at a certain angle to the polarization of the basic transverse waves. As the wave propagates, the type of its polarization will change, and at some distance, the wave will acquire circular polarization. This distance determines the length of the buffer rod so that the trajectory of particles on its second end surface will represent a circle. Therefore, the excitation of CPAW in the acoustic duct attached to the buffer rod second end is obtained. However, this design has some disadvantages, in particular, its large overall dimensions.

**Figure 4:**
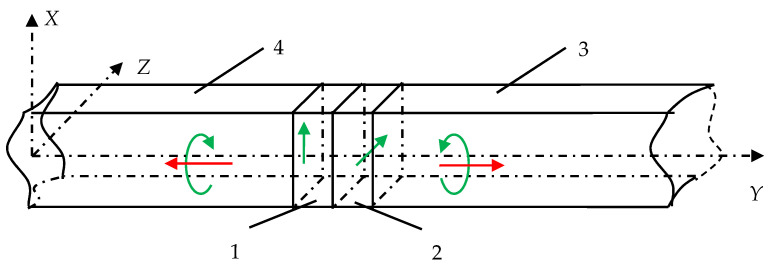
The new design of acoustic transducer for direct radiation of CPAW.

To eliminate these shortcomings, the authors of the present paper have developed a new design of an acoustic transducer, which makes it possible to radiate the CPAW directly, as shown in Figure 4.

Here, two piezoelectric plates 1 and 2 are in acoustic contact with each other. Plate 1 performs shear vibrations in the *X*-axis direction, thereby exciting transverse vibrations. Similarly, the piezoelectric plate 2 excites shear vibrations in the orthogonal *Z*-axis direction. The outer edges of both plates are acoustically connected to the acoustic ducts 3, so that the polarizations of the emitted ultrasonic waves are mutually orthogonal. The piezoelectric plate’s thickness should provide wave propagation time equal to a quarter of the oscillation period. At the given frequency, this ensures the superposition of two waves of orthogonal polarization at the time when their phase difference is equal to π⁄2. Therefore, the total circularly polarized wave propagates into the acoustic duct. Due to the symmetry of the transducer construction, wave radiation is carried out by both outer surfaces of the piezoelectric plates. The choice of the material and its cut for both piezoelectric plates are based on the criterion of the possibility to excite a pure transverse wave. Also, it is desirable to have the material with the high electromechanical coupling coefficient to increase the signal-to-noise ratio.

**Figure 5:**
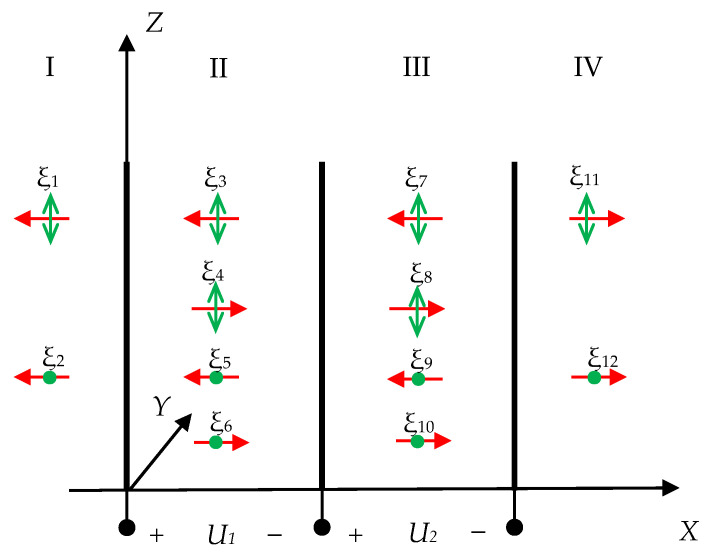
The multilayer system for direct CPAW radiation.

At the initial stage, the theoretical analysis of the transducer operation mode was performed. Therefore, the problem of propagation of two types (*X*-axis and *Z*-axis polarization) of transverse waves was solved for a multilayer system, which is shown in Figure 5.

The system consists of four layers I–IV, where I and IV are isotropic acoustic ducts with acoustic resistances *z*_1_, *z*_4_, respectively; II and III are piezoelectric plates *P*_1_ and *P*_2_ with acoustic resistances z_2_, z_3_, respectively; ξ_1_ − ξ_12_ are the propagating waves, where the horizontal arrow is the propagation direction and the vertical arrow and the dot is the polarization direction (particles displacement of the medium); *U*_1_, *U*_2_ are the electrical voltages applied to the piezoelectric plates’ electrodes.

The propagation of waves into each layer is described by a one-dimensional equation, which solution, in the general case, is a set of two oncoming waves. Therefore, it is necessary to find the unknown components ξ*_i_*_0_. Here, the boundary conditions consist of the displacement vector continuity and the stress’ normal components equality at the layers’ boundaries:

(10) $$\{\begin{array}{c} \xi_{1}{|}_{x=0}=\xi_{3}+\xi_{4}{|}_{x=0}\\ \xi_{2}{|}_{x=0}=\xi_{5}+\xi_{6}{|}_{x=0}\\ \xi_{3}+\xi_{4}{|}_{x=d_{1}}=\xi_{7}+\xi_{8}{|}_{x=d_{1}}\\ \xi_{5}+\xi_{6}{|}_{x=d_{1}}=\xi_{9}+\xi_{10}{|}_{x=d_{1}}\\ \xi_{7}+\xi_{8}{|}_{x=d_{1}+d_{2}}=\xi_{11}{|}_{x=d_{1}+d_{2}}\\ \xi_{9}+\xi_{10}{|}_{x=d_{1}+d_{2}}=\xi_{12}{|}_{x=d_{1}+d_{2}}\\ \sigma_{12}^{I}{|}_{x=0}=\sigma_{12}^{\mathrm{II}}{|}_{x=0}\\ \sigma_{13}^{I}{|}_{x=0}=\sigma_{13}^{\mathrm{II}}{|}_{x=0}\\ \sigma_{12}^{\mathrm{II}}{|}_{x=d_{1}}=\sigma_{12}^{\mathrm{III}}{|}_{x=d_{1}}\\ \sigma_{13}^{\mathrm{II}}{|}_{x=d_{1}}=\sigma_{13}^{\mathrm{III}}{|}_{x=d_{1}}\\ \sigma_{12}^{\mathrm{III}}{|}_{x=d_{1}+d_{2}}=\sigma_{12}^{\mathrm{IV}}{|}_{x=d_{1}+d_{2}}\\ \sigma_{13}^{\mathrm{III}}{|}_{x=d_{1}+d_{2}}=\sigma_{13}^{\mathrm{IV}}{|}_{x=d_{1}+d_{2}} \end{array}$$

The mechanical stress components in isotropic medium are determined by,

(11) $$\sigma_{ij}=\left(\lambda +2\mu \right)\frac{\partial \xi_{i}}{\partial x_{j}},$$

where λ and µ are the Lame’s elastic moduli.

For the piezoelectric medium, the mechanical stress and the electric induction vector are described by a system of piezoelectric effect equations, which in the matrix notation have the form,

(12) $$\{\begin{array}{c} \sigma_{j}=C_{jq}^{E}u_{q}-e_{jk}E_{k}\\ D_{i}=e_{iq}u_{q}+\varepsilon_{ik}^{u}E_{k} \end{array},$$

where $\sigma_{j}$ is the mechanical stress component; $C_{jq}^{E}$—modulus of elasticity at constant electric field; $u_{q}$—elastic deformation; $e_{jk}$—piezoelectric constant; $E_{k}$—electric field strength; $\varepsilon_{ik}^{u}=\varepsilon_{0}\left(\varepsilon_{ik}^{u}\right)\prime$—dielectric permittivity at constant deformation; $\varepsilon_{0}$—dielectric constant; ($\varepsilon_{ik}^{u}$)′—relative values of the components.

The *u_q_* and *E_k_* components can be expressed in terms of the desired wave amplitudes. By substituting (11), (12) in (10), a system of twelve equations for twelve unknown wave amplitudes can be obtained. When using piezo quartz as a transducer material, it is necessary to take into account the longitudinal component associated with the material anisotropy.

The solution of this system of equations makes it possible to determine the motion characteristics of the particles located on the surface of the piezoelectric plates.

**Figure 6:**
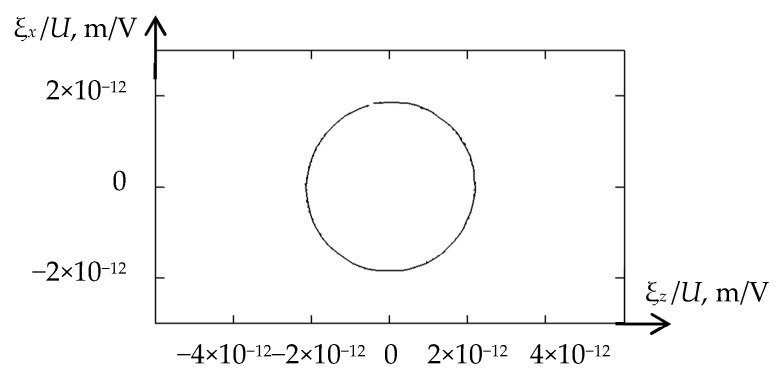
The trajectory of particle motion in radiated wave.

Figure 6 shows the trajectory of particle motion for the case of equal applied voltages *U*_1_ = *U*_2_ and acoustic ducts made of fused quartz (*z*_1_ = *z*_4_).

As can be seen, the calculated trajectory has an almost ideal shape of a circle, which theoretically confirms the efficiency of the proposed design of the acoustic transducer for direct CPAW radiation.

According to numerical modeling results, the directions of particles’ rotation for waves propagating in opposite directions are different. An important note is that the acoustic impedances ρc of the acoustic ducts, which are the transducer loads, must be equal.

### 3.2. Experimental Research 

**Figure 7:**
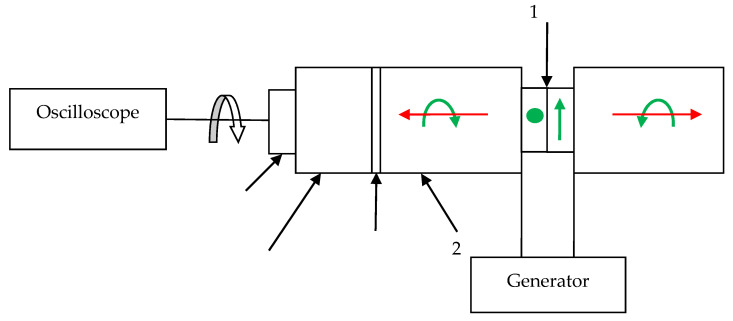
The scheme of experimental setup for CPAW transducer.

To verify the theoretical assumption and calculation estimation of the circular character of particle motions in the acoustic wave, radiated by the developed transducer, the experimental studies were carried out. The experimental setup is shown in Figure 7.

The optimal frequency of the emitting transducer 1 was chosen equal to 2.2 MHz according to the results of numerical simulation, based on the criterion to obtain the particle trajectory closest to a circle.

Acoustic waves were received from the opposite end of the acoustic duct 2 by a piezoelectric plate 3, which detects only linearly polarized waves, through a buffer rod 4. Since the position of the receiving plate should be changed during the experiment, an epoxy without a hardener was chosen as the contact layer 5 because of the physical properties to transmit shear vibrations. Therefore, if the amplitude of the detected signal does not depend on the orientation of the receiving plate, the received acoustic wave has a circular polarization.

**Figure 8:**
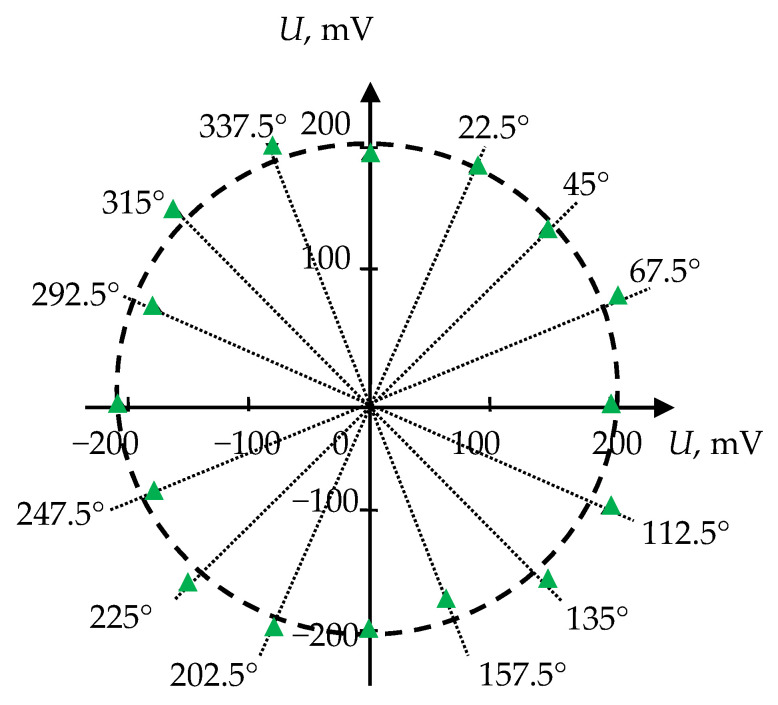
The experimental results: the dependence of the received signal amplitude on the angle of rotation of the receiving piezoelectric plate.

During the experiment, the dependence of the amplitude of the received signal on the angle of rotation of the receiving piezoelectric plate was determined in the range of angles 0–360° for the initial orientation direction, taken as 0, at the outer ends of both acoustic ducts. The experimental results are shown in Figure 8. The experimental points are marked with triangles, and the length of the radius vector corresponds to the amplitude of the received signal at the fixed angle.

The obtained angular distribution of the amplitude has the shape of a circle. The deviation from the correct circle form can be related to a change of the acoustic contact quality during measurements and to the technological error in processing and manufacturing of piezoelectric plates, in terms of their geometry and, accordingly, characteristics. In general, the experimental results indicate that the nature of the particle motion in the emitted wave is close to circular. Therefore, the carried-out theoretical, computational, and experimental studies have shown the possibility of direct emission of CPAW with a designed construction of the acoustic transducer.

## 4. Experimental Research

The next stage in the complex research of a new concept of a solid-state acoustic RRS was the experimental studies of the developed and manufactured SE on CPAW to confirm its efficiency.

**Figure 9:**
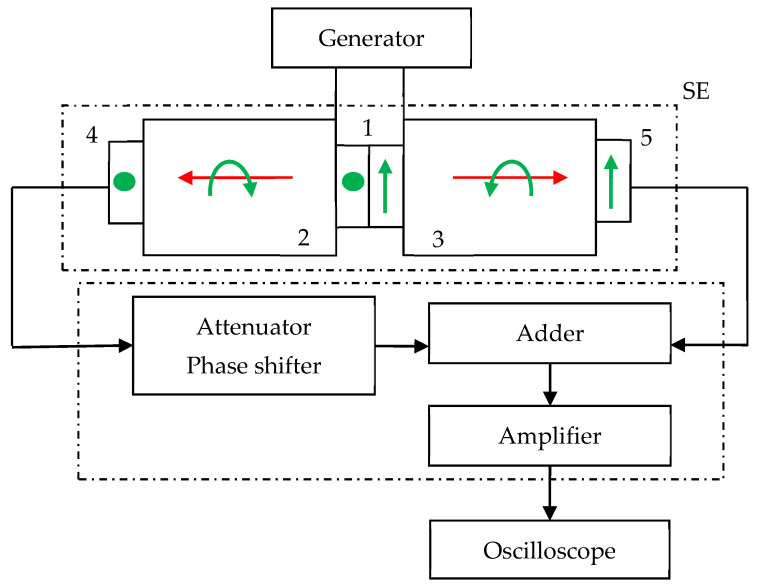
The scheme of the test model of the RRS SE on CPAW.

As mentioned above, to detect an informative signal (which is a change in the phase velocity of the emitted CPAW) proportional to the rotation rate, the pulse-phase method was chosen. For its implementation, the scheme for a test model of the RRS shown in Figure 9 was suggested. At the same time, the differential principle of obtaining an informative signal is realized, which makes it possible to exclude the influence of external factors, such as temperature changes, and doubles the sensitivity of the method.

The generator of high-frequency impulses generates a harmonic electrical signal of a certain amplitude and frequency, which is fed to the CPAW transducer 1. The transducer 1 excites two circularly polarized acoustic waves with an amplitude that changes according to the harmonic law. These waves propagate in opposite directions in the acoustic ducts 2 and 3. At the outer ends of the acoustic ducts, the receiving piezoelectric shear plates 4 and 5 are placed, which transform the received elastic vibrations into an electrical signal, and its total amplitude displays on the oscilloscope screen.

**Figure 10:**
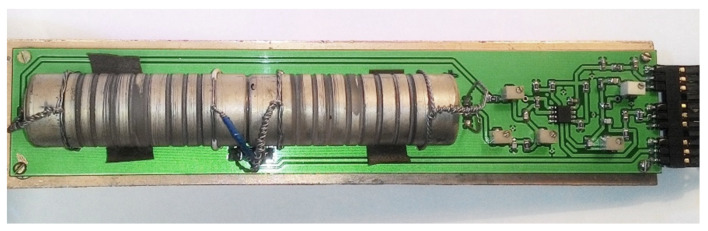
The RRS SE on CPAW.

**Figure 11:**
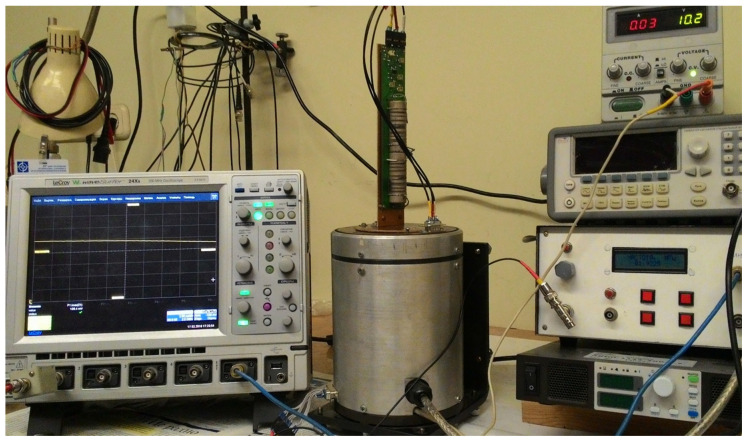
The experimental setup with test model of the RRS SE on CPAW.

The RRS SE on CPAW and the experimental setup are given in Figure 10 and Figure 11, correspondingly.

According to the earlier calculations [16,17], the directions of particle motions in the emitted waves are the opposite. As a result, under rotation of the acoustic lines about the *X*-axis of CPAW propagation, the phase velocity of one of them receives a positive addition *V*_1_ = *V*_0_/((1 + Ω/ω)), and the other—the negative one *V*_2_ = *V*_0_/((1 − Ω/ω)). Due to a change of the waves’ propagation time to the receiving piezo plates, the phases of the received signals change as well, which in turn affects the amplitude of the total signal, which is the informative parameter related to the rotation rate of the entire system.

Let us estimate the change of the output voltage due to rotation. At the initial moment, a harmonic electrical signal *U*in = *U*_0_ cos(ω*t*) is applied to the emitting electrodes, which leads to the radiation of CPAW with particle displacement ξ = ξ_0_ cos(ω*t*). After the wave has passed a distance equal to the length of the acoustic duct *L*, it acquires a phase shift φ_0_ = *kL* = (ω/*V*_0_)*L* = ωτ_0_, where τ_0_ is the time it takes for the wave to travel the distance *L*. Then the displacement is determined as ξ = ξ_0_ cos(ω(*t −* τ_0_)), and the received voltage is *U* = *U*_0_ cos(ω(*t −* τ_0_)).

**Figure 12:**
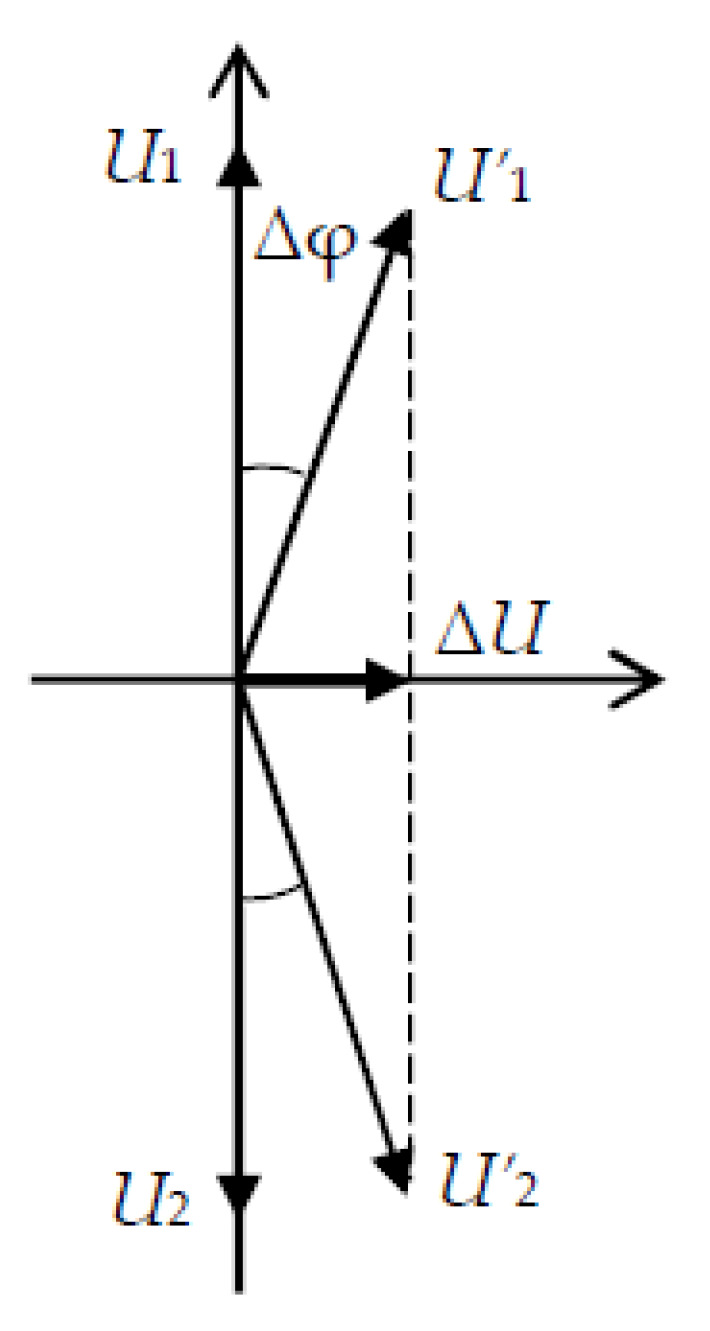
The informative signal Δ*U* proportional to the rotation velocity Ω (where *U*_1_, *U*_2_ are the output voltages without rotation, *U’*_1_, *U’*_2_ are the output voltages under rotation)**.**

In the case when the acoustic duct is under rotation with an angular velocity Ω, when the wave propagates over a distance *L*, the phase shift is determined by φ = *kL* = (ω(1 ± *W*)/*V*_0_)*L* = φ_0_ ± τΩ. Thus, the phase of the received signal has an additional phase shift Δφ = τΩ, which leads to a change in the amplitude of the received voltage Δ*U*, as shown in Figure 12. For *U*_1_ = *U*_2_, *U’*_1_ = *U’*_2_ = *U’*, so that Δ*U = 2U’* sin Δφ ≈ *2U’* Δφ for small Δφ. Thus, the informative signal, which is the change of the output electric voltage, is proportional to the rotation rate Δ*U = 2U’* τΩ.

To ensure the minimum amplitude of the total signal in the absence of rotation, that is, to add the received signals in opposite phases, the receiving piezoelectric plates 4 and 5 are chosen to detect only shear oscillations. The directions of displacement sensitivity of these plates are mutually orthogonal. For equal lengths of acoustic ducts 2 and 3, it makes possible to compensate the two received electrical signals at the operating frequency.

For the amplitude equalization and phase adjustment of the received signals, an attenuator, and a phase shifter are required, respectively. To minimize the effect of broadband electromagnetic interference on the output signal, a narrowband frequency-selective device is used.

## 5. Experimental Results

At the next stage, the estimation was made of the magnitude of the output voltage change, due to the SE RRS rotation around the axis of CPAW propagation.

Experimental studies of the test model were carried out by measuring the dependence of the amplitude of the received signal on the SE rotation rate around the sensitivity axis. The test model was placed on a measuring plate of the special centrifuge [21], which rotation parameters can be set precisely via the PC interface.

To determine a signal level the oscilloscope was used, which provides measuring the amplitude in a given time interval.

**Figure 13:**
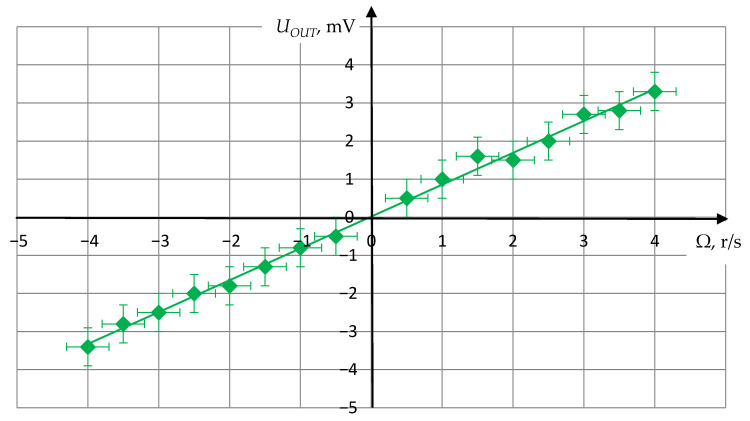
The experimental dependence of the output voltage on the rotation rate of RRS SE on CPAW.

The rotation rate of the centrifuge was set discretely with a step of 0.5 r/s. The experimental results obtained on the test setup shown in Figure 11 are presented graphically in Figure 13.

As seen from Figure 13, the obtained dependence has a linear character, which corresponds to the theoretical estimation in Section 4, and makes it possible to determine the scale factor between the angular velocity and the output voltage. It is important to note that the scale factor is constant regardless of the rotation direction. The characteristics slope is determined by the gain of the receiving path.

Thus, the results of full-scale tests of the developed RRS SE model, carried out on an experimental setup, confirmed the reliability of the theoretical and computational studies, described in Section 4, and showed the possibility of design a solid-state acoustic RRS based on CPAW.

## 6. Discussion

When studying the features of the BAW propagation in the direction orthogonal to the axis of medium rotation the following conclusions can be made:

1. As shown by the theoretical analysis of the propagation of bulk shear waves along the rotation axis, the phase velocity of a circularly polarized acoustic wave depends on the rotation rate.
2. Based on the revealed effects, the change in the phase velocity of the circularly polarized acoustic wave is suggested to be an informative parameter for rotation rate solid-state acoustic sensors design.
3. Based on the results of theoretical analysis, a new design of a piezoelectric transducer for direct radiation of circularly polarized acoustic wave was offered and designed. The performed computational modeling and experimental research confirmed the circular nature of the particle motion in the radiated wave.
4. Based on the results of theoretical analysis, the new physical concept of the rotation rate sensor on the circularly polarized acoustic wave was developed, experimental studies of the test model were carried out, which confirmed its efficiency. The sensitivity of the developed acoustic sensor was estimated to be 340 μV/r/s.
5. The theoretical assumptions, as well as the proposed concept for acoustic solid-state sensor design are in agreement with the experimental results demonstrating the linear-type dependence of the informative parameter on the angular velocity of medium rotation.
6. The level of the informative signal, obtained experimentally, shows a high correlation with the previously determined theoretical sensitivity level of the proposed model of the rotation rate sensor based on circularly polarized acoustic waves.

## 7. Patents

1. Lutovinov A.I.; Lukyanov D.P.; Peregudov A.N.; Pozhenskaya A.A.; Shevelko M.M. Method of measurement of angular rate and sensitive element of gyroscope based on it. 27 June 2014, Patent of Russian Federation *№ 2520949*.
2. Lutovinov A.I.; Pozhenskaya A.A.; Peregudov A.N.; Shevelko M.M. Piezoelectric transducer. 10 June 2014, Patent of Russian Federation *№ 2529824*.

## Data Availability

Data is contained within the article.
